# DNA methylation regulates B cell activation via repressing Pax5 expression in teleost

**DOI:** 10.3389/fimmu.2024.1363426

**Published:** 2024-02-09

**Authors:** Yuan Shi, Zhuo Zhu, Qiuxuan Chen, Xinhua Chen

**Affiliations:** ^1^ State Key Laboratory of Mariculture Breeding, Key Laboratory of Marine Biotechnology of Fujian Province, College of Marine Sciences, Fujian Agriculture and Forestry University, Fuzhou, China; ^2^ Southern Marine Science and Engineering Guangdong Laboratory (Zhuhai), Zhuhai, China; ^3^ Fuzhou Institute of Oceanography, Fuzhou, China

**Keywords:** DNA methylation, Pax5, IgM, B cells, large yellow croaker (*Larimichthys crocea*)

## Abstract

In mammals, the transcription factor Pax5 is a key regulator of B cell development and maturation and specifically expressed in naive/mature B cells but repressed upon B cell activation. Despite the long-standing proposal that Pax5 repression is essential for proper B cell activation, the underlying mechanisms remain largely elusive. In this study, we used a teleost model to elucidate the mechanisms governing Pax5 repression during B cell activation. Treatment with lipopolysaccharide (LPS) and chitosan oligosaccharide (COS) significantly enhanced the antibody secreting ability and phagocytic capacity of IgM^+^ B cells in large yellow croaker (*Larimichthys crocea*), coinciding with upregulated expression of activation-related genes, such as Bcl6, Blimp1, and sIgM, and downregulated expression of Pax5. Intriguingly, two CpG islands were identified within the promoter region of Pax5. Both CpG islands exhibited hypomethylation in naive/mature B cells, while CpG island1 was specifically transited into hypermethylation upon B cell activation. Furthermore, treatment with DNA methylation inhibitor 5-aza-2’-deoxycytidine (AZA) prevented the hypermethylation of CpG island1, and concomitantly impaired the downregulation of Pax5 and activation of B cells. Finally, through *in vitro* methylation experiments, we demonstrated that DNA methylation exerts an inhibitory effect on promoter activities of Pax5. Taken together, our findings unveil a novel mechanism underlying Pax5 repression during B cell activation, thus promoting the understanding of B cell activation process.

## Introduction

The immune system is an intricate network of cells and molecules that defends the organisms against various pathogens, including bacteria, parasites and viruses. B cells play a crucial role in adaptive immunity within the diverse components of the immune system. During mammalian hematopoiesis, hematopoietic stem cells (HSCs) residing in the bone marrow initially differentiate into lymphoid-primed multipotent progenitors (LMPPs), which subsequently produce common lymphoid progenitors (CLPs), with potential of generating B cells, T cells, and natural killer cells (NK) (1). In terms of B cell lineage commitment, CLPs undergo a series of differentiation steps including B-progenitors (pro-B), precursor B cells (pre-B) and immature B cells before ultimately matured into functional B cells (2, 3). Upon antigen stimulation, naive/mature B cells initiate the germinal center (GC) reaction and differentiate into antibody secreting plasma cells (4).

Notably, the transcription factor Pax5 plays crucial roles during the development and maturation of B cells (5, 6). Homozygous Pax5 mutation in mice inhibits B cell development at early pro-B cell stage and impairs the expression of maturation marker genes, such as CD19, CD25, and BP-1 (7). Furthermore, studies have demonstrated that Pax5 not only promotes the expression of B cell genes, but also inhibits the expression of other lineage genes in B cells to ensure the proper differentiation of B cells (8, 9). Moreover, Pax5 is also involved in the fate maintenance of mature B cells. Transplantation of Pax5 mutated mature B cells into T-cell-deficient mice has rescued the T lymphopoiesis in the recipient mice, indicating that Pax5 deletion can induce transdifferentiation from B cells into T cells (10).

Despite its indispensable role during B cell development and maturation, the expression of Pax5 is repressed during B cell activation, accompanied by the suppression of the mature B cell gene expression and the B cell receptor (BCR) signaling (11, 12). The repression of Pax5 and mature program has long been postulated to be essential for appropriate B cell activation and plasma cell differentiation (13, 14). A recent study suggested that Pax5 repression is not necessary for plasma cell development or antibody secretion, however, the repression of Pax5 is required for optimal IgG production and accumulation of long-lived plasma cells (15). Despite the well-recognized suppression of Pax5 in activated B cells, the underlying mechanisms remain elusive.

Teleost are the most ancient bony vertebrates with bona fide B cells. Among teleost, IgM^+^ B cells represent a predominant subtype and are widely distributed in systemic immune organs/tissues, such as head kidney, spleen, and blood (16, 17). To investigate the mechanisms underlying Pax5 repression during B cell activation in the ancient vertebrates, we used large yellow croaker (*Larimichthys crocea*), an economically important marine fish in China and East Asia, as a model. We established an efficient strategy to activate IgM^+^ B cells of large yellow croaker, and found that the activation of IgM^+^ B cells is accompanied with a transition from hypomethylation into hypermethylation of a CpG island located within the promoter region of Pax5. Inhibition of the DNA methylation impaired the downregulation of Pax5 and B cell activation. Our findings provide valuable insights into the mechanisms underlying Pax5 repression during B cell activation.

## Methods

### Experimental fish

Large yellow croakers (51.3 ± 7.1 g) were obtained from a mariculture farm in Ningde, Fujian, China, and raised as previously described (18, 19). The fish were maintained at a temperature of 20 ± 2°C with a continuous flow of seawater and fed commercial feed pellets twice daily. After a 7-day acclimation period, healthy fish were used for the subsequent experiments. This study was conducted in strict accordance with the Regulations of the Administration of Affairs Concerning Experimental Animals established by the Fujian Provincial Department of Science and Technology. All surgeries were performed under Tricaine-S anesthesia, and every effort was made to minimize suffering.

### Leukocytes isolation

The primary head kidney leukocytes (PKLs) from large yellow croaker were prepared as previously described (20, 21). Briefly, the head kidney was aseptically collected from the freshly killed fish and gently passed through a 70-μm nylon mesh (BD) to obtain single-cell suspensions. The cell suspensions were washed twice with ice-cold DMEM containing 2% FBS (Life Technologies), 15 IU/ml heparin sodium, and 2% penicillin/streptomycin (Life Technologies). Each single-cell suspension was loaded onto a freshly prepared density gradient composed of 34%/51% Percoll (GE), followed by centrifugation at 650 × g for 30 min at 4°C. Subsequently, the PKLs at the gradient interface were collected and washed twice with DMEM before being resuspended in DMEM containing 2% FBS and 2% penicillin/streptomycin for subsequent experiments.

### Magnetic-activated cell separation

MACS was applied to sort IgM^+^ B cells from the PKLs as previously described, with minor modifications (17). Briefly, the monoclonal mouse anti-*Lc*IgM was conjugated with FITC and utilized to label the collected leukocytes (1 μg/ml) (22). After incubation at 4°C for 20 min, the cells were washed twice with sterile PBS, and resuspended in MACS buffer (PBS supplemented with 2 mM EDTA and 0.5% BSA, pH 7.2) at a concentration of 1 × 10^8^ cells/mL. Subsequently, 50 μl Anti-FITC MicroBeads (Miltenyi Biotec) were added into the buffer and incubated at 4°C for 15 min. After washing twice with sterile PBS, the cells were resuspended in 5 mL MACS buffer and loaded onto the LS columns, which were attached to the MACS separator (Miltenyi Biotec). The LS columns were then washed five times with the MACS buffer, and the flow-through was harvested for collection of unlabeled IgM^-^ cells. Finally, the LS columns were detached from the MACS separator, and 5 ml of MACS buffer was pipetted onto each column. The labeled IgM^+^ cells were collected by pushing the plunger into the column.

### Gene expression analysis by real-time quantitative PCR

After resting overnight, IgM^+^ B cells were stimulated with 20 μg/ml lipopolysaccharide (LPS, sigma) and chitosan oligosaccharide (COS, Solarbio Life Sciences), with or without the treatment of 50 μM 5-aza-2’-deoxycytidine (AZA, Selleck). TRIzol reagent (Invitrogen) was used for extraction of total RNA from cultured cells. The cDNA was synthesized using All-In-One 5× RT MasterMix (abm), and then was diluted 5 times in nuclease-free water for qPCR analysis. qPCR experiments were conducted on a QuantStudio 5 instrument (Thermo Fisher, USA) using the SYBR qPCR Master Mix (Vazyme). Specific primers for were designed based on the genome assembly of large yellow croaker (**Supplementary Table 1**) (23, 24). Secreted IgM (sIgM) and membrane-bound IgM (mIgM) were previously identified in our lab (25, 26). The relative gene expression level was determined by the 2^−ΔΔCt^ method (27), normalized against the reference gene *Lc*β-actin, and the fold change was calculated by comparison with the control group. All qPCR reactions were performed with three biological replicates.

### ELISA

The IgM concentration in the cell supernatant was quantified using a double antibody sandwich ELISA (28). Briefly, cultured IgM^+^ B cells were centrifuged at 550 × g for 5 min at 4°C and the cell-free supernatant was collected. The 96-well ELISA plate (Costar) was coated overnight with 100 μl of PBS containing 2.5 μg/ml rabbit anti-*Lc*IgM, and then blocked with 100 μl of PBST containing 1% BSA for 2 h at 37°C. After washing three times with PBST, 100 μl of sample diluted in PBS containing 1% BSA was added to each well. Following incubation at 37°C for 2 h, the plates were washed three times with PBST, and monoclonal mouse anti-*Lc*IgM (2.5 μg/ml) in PBS was then added, followed by an incubation at 37°C for 1 h. After washing three times with PBST, 100 μl of HRP-goat anti-mouse antibody was added and incubated at 37°C for 1 h. After washing three times with PBST, 100 μl of TMB substrate solution (Solarbio) was added per well and incubated at 37°C for 10 min, then 100 μl of stopping buffer was added and the OD450 was examined by a plate reader (Promega). Antibody reactivity was calculated after subtraction of background (OD450 of culture medium coated plates).

### Phagocytosis assay

Phagocytosis assay was performed as previously described, with minor modifications (29). The cultured IgM^+^ B cells were co-incubated with 1.0-μm fluorescent beads (Invitrogen) at a cell/bead ratio of 1:10 for 3 h at 28°C. Then cell suspensions were loaded onto a cushion of 3% BSA in PBS supplemented with 4.5% d-glucose and then centrifuged at 550 × g for 10 min at 4°C. The resultant cells were washed with PBS for two times, and then used for further flow cytometry analysis (BD) and imaged under a laser scanning confocal microscope (Leica).

### CpG islands prediction and bisulfite sequencing PCR

The CpG islands in the promoter region (2 kb upstream of transcription start site) of Pax5 were predicted using the online software MethPrimer (30), and the BSP primers were designed surrounding the CpG islands (**Supplementary Table 1**). Genomic DNA from cultured IgM^+^ B cells under different treatments was extracted with the Tissue DNA Kit (Omega biotek) and then subjected to bisulfite conversion using the EZ DNA Methylation-Gold Kit (Zymo Research) following the manufacturer’s instructions. Subsequently, BSP was performed as previously described with minor modifications (31). Briefly, touchdown PCR was conducted with the bisulfite converted genomic DNA and BSP primers: initial denaturation step (95°C 10 min), 30 cycles [95°C 30 s, 60-45°C (with gradual decrease of 0.5°C per cycle) 30 s, 72°C 1 min], 20 cycles (95°C 30 s, 45°C 30 s, 72°C 1 min), final extension step (72°C 10 min). The amplified products were purified after gel electrophoresis, and cloned into pEASY-T1 vectors (Trans Gen Biotech). Ten clones for each gene were collected for sequencing with three biological replicates for each treatment. QUMA software was utilized for the analysis and quantification of DNA methylation level (32).

### Dot blot assay

Dot blot assay was used to analyze the global 5-methylcytosine (5mC) level as previously reported (33). Genomic DNA from cultured large yellow croaker IgM^+^ B cells under different treatments was extracted with the Tissue DNA Kit (Omega biotek). 500 ng of genomic DNA was transferred to a Nylon membrane (Beyotime), and rabbit anti-5mC antibody (1: 1000; Cell Signaling Technology) was used to detect the global 5mC level of the samples.

### Luciferase reporter assay

The promoter region of Pax5 was amplified and cloned into the pGL3-basic vector (**Supplementary Table 1**). The resulting recombinant plasmids were *in vitro* methylated using CpG Methyltransferase M.SssI (New England BioLabs) following the manufacturer’s instructions. Methylation of recombinant plasmids was confirmed by digestion with the *Hpa*II enzyme, which specifically digests unmethylated DNA. HEK293T cells were seeded in 24-well plates, with 1 × 10^5 cells per well, and cultured in DMEM supplemented with 10% FBS and 1% penicillin/streptomycin at 37°C with a supply of 5% CO_2_. After culturing overnight, each well is transfected with 500 ng methylated or unmethylated plasmids using Lipo8000 (Beyotime), and 50 ng pRL-TK plasmid was used as internal control. After 48 hours of culture, luciferase activity was measured with Dual-Luciferase Reporter Assay System (Promega) following the manufacturer’s instructions. All assays were performed with three biological replicates.

### Statistical analysis

Statistical analyses were performed using GraphPad Prism 9 software. Data were presented as the mean ± standard deviation (SD). Group comparisons were assessed using unpaired Student’s *t* test, and statistical significance was defined as *p* < 0.05.

## Results

### Enrichment of IgM^+^ B cells via MACS

**Figure 1:**
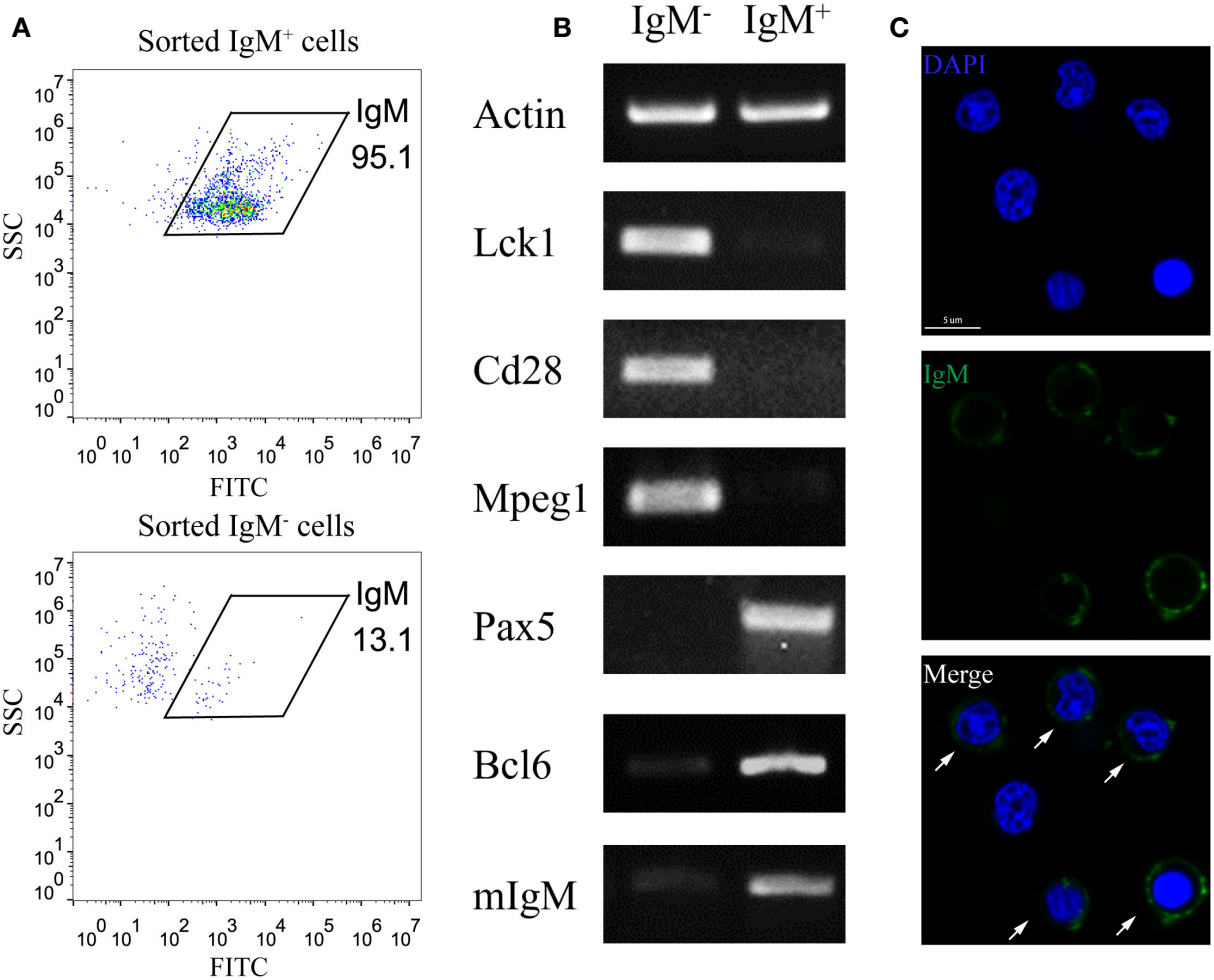
Enrichment of IgM^+^ B cells through MACS. **(A)** FACS analysis showed the IgM^+^ cell percentage of MACS sorted IgM^+^ cells and IgM^-^ cells. **(B)** RT-PCR showed the expression pattern of T cell marker genes (Lck1, Cd28), macrophage marker gene (Mpeg1) and B cell marker genes (Pax5, Bcl6, mIgM) in MACS sorted IgM^+^ cells and IgM^-^ cells. **(C)** Confocal microscopy showed the majority of MACS sorted IgM^+^ cells are labelled by anti-IgM antibody. Arrows indicate the anti-IgM antibody labelled cells. Scale bar, 5 μm.

To minimize the interference of other cell types in studying B cell activation, we utilized the MACS method to enrich the IgM^+^ B cell from the PKLs of large yellow croaker. Flow cytometry analysis revealed the purity of MACS sorted IgM^+^ B is above 95% (**Figure 1A**). RT-PCR showed that MACS sorted IgM^+^ cells exhibited high expression levels of B cell marker genes (Pax5, Bcl6, mIgM), while genes related to T cells (Lck1, Cd28) and macrophages (Mpeg1) were not detected. In contrast, MACS sorted IgM^-^ cells showed apparent expression of T cell and macrophage related genes (**Figure 1B**). Furthermore, immunofluorescence microscopy confirmed that the majority of the sorted IgM^+^ cells are labelled with anti-*Lc*IgM antibody (**Figure 1C**). These results indicated that MACS is an efficient approach to enrich IgM^+^ B cells.

### Efficient activation of IgM^+^ B cells through LPS and COS treatment

**Figure 2:**
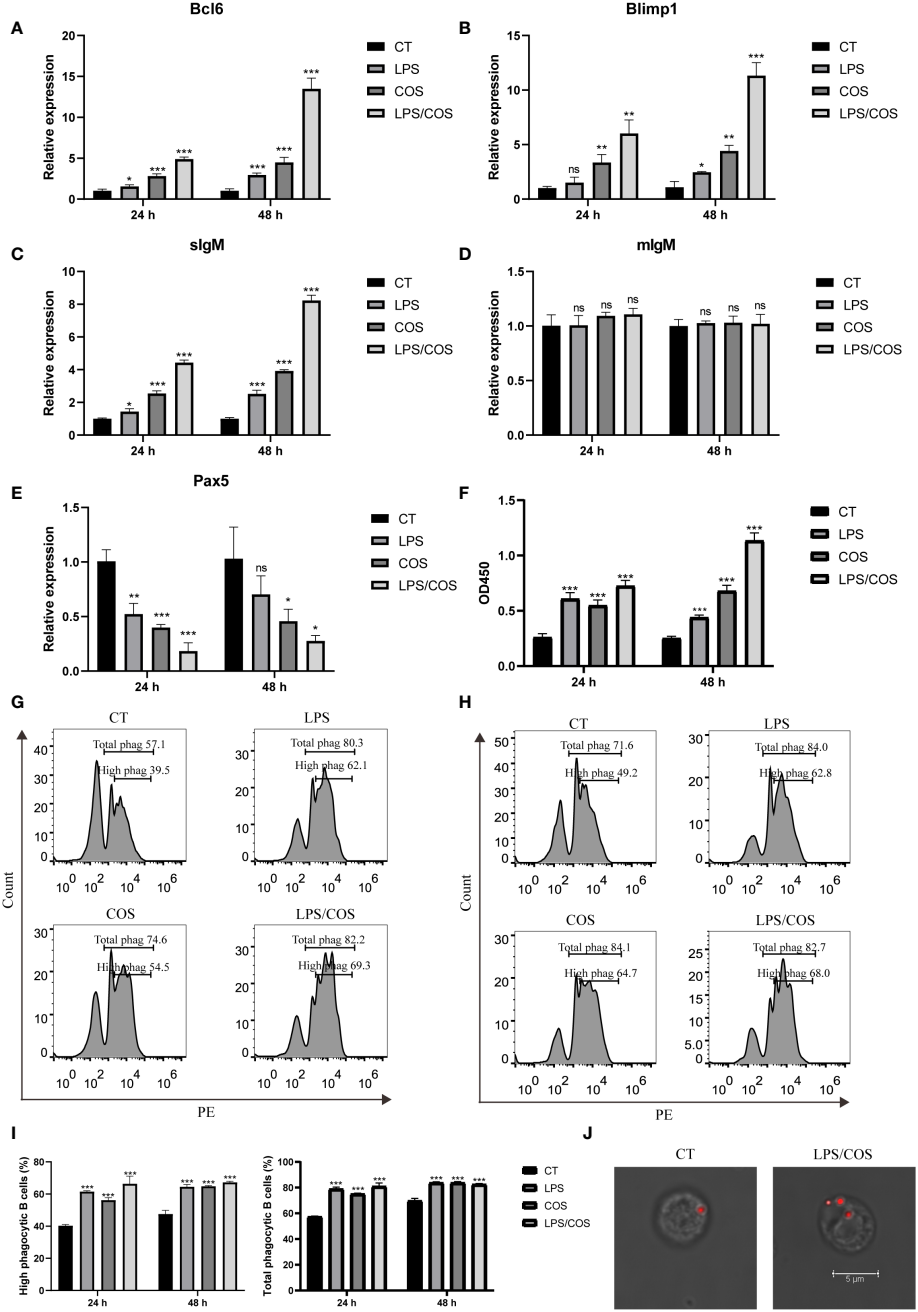
LPS and COS treatment induced efficient activation of IgM^+^ B cells. **(A–E)** qPCR showed the expression pattern of Bcl6, Blimp1, sIgM, mIgM and Pax5 in IgM^+^ B cells after treatment of LPS and COS. **(F)** ELISA analysis of the concentration of secreted IgM in the culture medium of IgM^+^ B cells stimulated by LPS and COS. **(G, H)** Flow cytometry analysis showed the percentage of phagocytic B cells at 24 hours **(G)** and 48 hours **(H)** after treatment of LPS and COS. **(I)** Statistical results of phagocytic B cells in **(G, H)**. **(J)** Representative confocal microscopy validated the phagocytic ability of B cells was increased after treatment of LPS and COS. *P <0.05, **P <0.01, ***P <0.001, ns: not significant.

For the activation of IgM^+^ B cells, we stimulated the IgM^+^ B cells with LPS and COS, which have been reported to activate B cells and PKLs in teleost (19, 34). qPCR analysis showed that treatment with LPS or COS respectively could upregulate the expression level of the genes related to B cell activation, such as Bcl6, Blimp1, and sIgM, and the enhancement effect is more significant with combined treatment of LPS and COS, while the expression level of mIgM remain unchanged (**Figures 2A–D**). Furthermore, the antibody secreting ability of IgM^+^ B cells was increased upon LPS and COS treatment (**Figure 2F**). In addition to their adaptive immune activities, teleost B cells also exhibit certain innate immune activities, such as phagocytic and bactericidal abilities (35). We thus examined the phagocytic capacity of IgM^+^ B cells after LPS/COS treatment. Flow cytometry analysis revealed a low PE^+^ population and a high PE^+^ population, which represented low phagocytic B cells and high phagocytic B cells, respectively (**Figures 2G–I**). We found that the proportion of both high phagocytic and total phagocytic IgM^+^ B cells is enhanced upon LPS and COS treatment. Furthermore, immunofluorescence microscopy confirmed that LPS and COS treatment increased the number of internalized beads in B cells (**Figure 2J**). These results indicated that the treatment with LPS and COS could efficiently activate both the adaptive and the innate immune activities of IgM^+^ B cells.

### BSP analysis of methylation level of CpG islands in Pax5 promoter region

**Figure 3:**
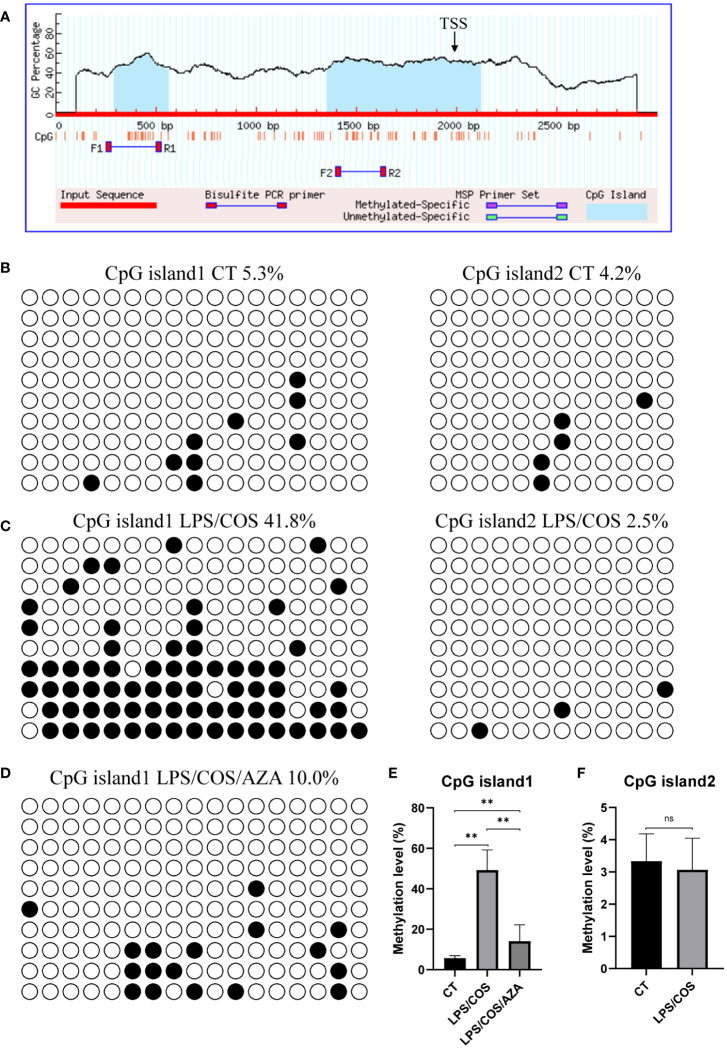
LPS/COS treatment induced hypermethylation of CpG island1 in Pax5 promoter region. **(A)** Two CpG islands were identified in promoter region of Pax5. TSS, transcription start site. **(B, C)** BSP analysis of DNA methylation level in CpG island1 and CpG island2 in naive/mature **(B)** and LPS/COS treated **(C)** IgM^+^ B cells. **(D)** BSP analysis of DNA methylation level in CpG island1 after LPS/COS/AZA treatment. **(E, F)** Statistical results of DNA methylation level of CpG island1 and CpG island2 in **(B–D)**. **P <0.01, ns: not significant.

Notably, we found that the expression of Pax5 is downregulated after LPS and COS treatment, indicating that Pax5 repression during B cell activation is conserved in teleost and mammals (**Figure 2E**). DNA methylation in the form of 5-methylcytosine (5mC) is a classic epigenetic modification that usually occurs within the CpG islands located in the gene promoter region. High level of 5mC DNA methylation in the promoter region is generally associated with transcriptional repression and plays a crucial role in various biological processes, such as development, homeostasis, and immune response (36, 37). To investigate whether the expression level of Pax5 is regulated by DNA methylation, we cloned and analyzed the promoter region of Pax5 by MethPrimer. Two CpG islands were identified in the promoter region of Pax5 (**Figure 3A**). The CpG island1 is located between nucleotides -1706 to -1437, and CpG island2 located between nucleotides -643 to +123. We then performed BSP analysis to examine the DNA methylation levels of these two CpG islands both before and after B cell activation. The results showed that both CpG island1 and CpG island2 are hypomethylated before B cell activation, which is corresponding to the high expression of Pax5 in naive/mature B cells (**Figures 3B, E, F**). After being stimulated by LPS and COS, the CpG island1 was transited into hypermethylation, while the CpG island2 remained hypomethylated (**Figures 3C, E, F**). These results suggested that the hypermethylation at CpG island1 may be related to the repression of Pax5 expression during B cell activation.

### Inhibition of DNA methylation impaired Pax5 repression and B cell activation

**Figure 4:**
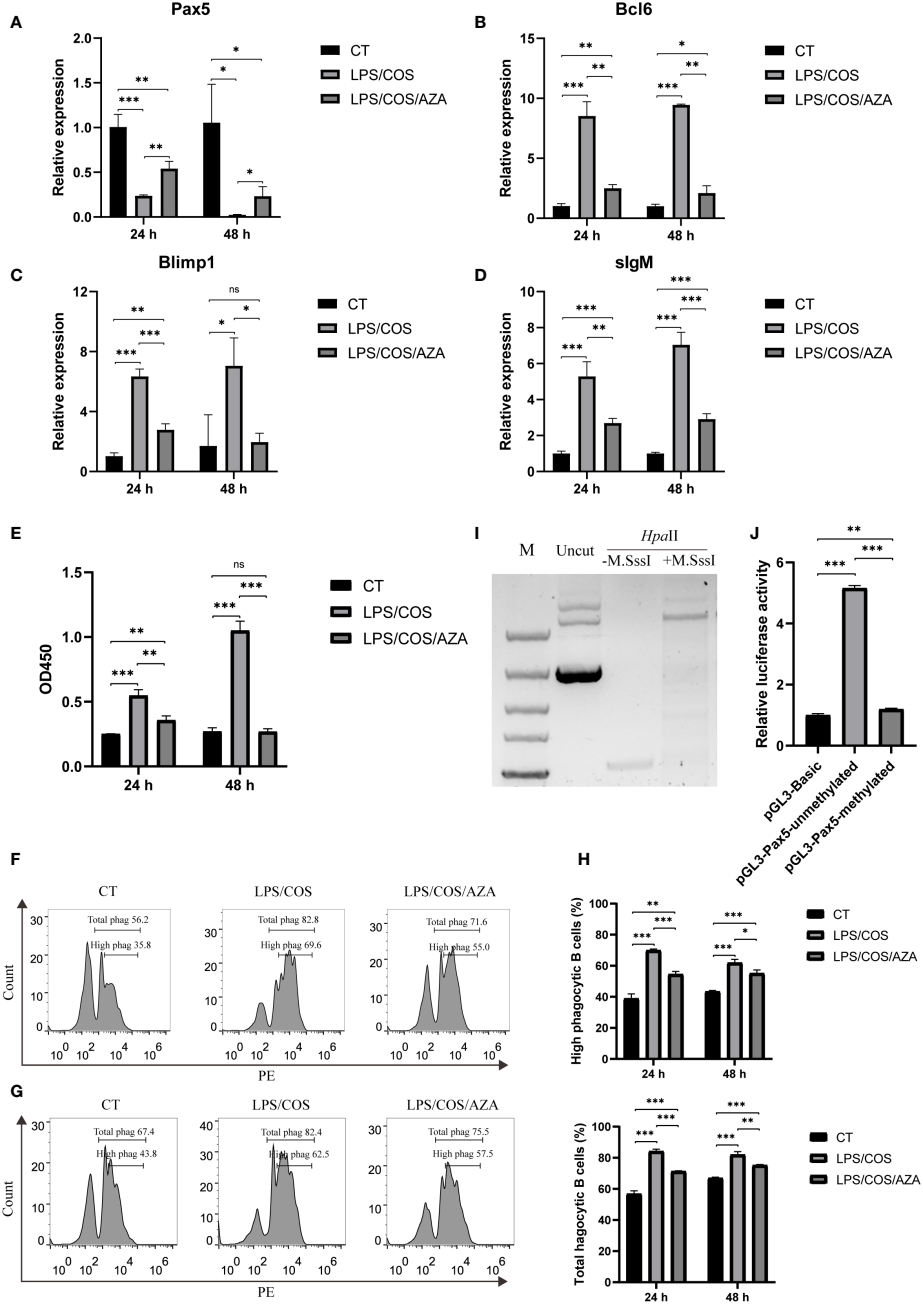
Inhibition of DNA methylation impaired downregulation of Pax5 and B cell activation. **(A–D)** qPCR showed the expression pattern of Bcl6, Blimp1, sIgM and Pax5 in IgM^+^ B cells after treatment of LPS/COS and LPS/COS/AZA. **(E)** ELISA analysis of the concentration of secreted IgM in the culture medium of IgM^+^ B cells after treatment of LPS/COS and LPS/COS/AZA. **(F, G)** Flow cytometry analysis showed the percentage of phagocytic B cells at 24 hours **(F)** and 48 hours **(G)** after treatment of LPS/COS and LPS/COS/AZA. **(H)** Statistical results of phagocytic B cells in F and G. **(I)** *in vitro* enzymatic digestion of M.SssI treated or not treated pGL3-Pax5 by *Hpa* II, which only digest unmethylated DNA. M.SssI treated group prevented the digestion by *Hpa*II, indicating the high efficiency of *in vitro* DNA methylation. **(J)** Luciferase assays showed the promoter activities of methylated or unmethylated pGL3-Pax5, pGL3-Basic was used as the negative control. *P <0.05, **P <0.01, ***P <0.001, ns: not significant.

We then used 5-aza-2’-deoxycytidine (AZA), a specific inhibitor of DNA methyltransferase, to investigate the role of DNA methylation during B cell activation. We found that the AZA treatment inhibited the hypermethylation of CpG island1 during B cell activation (**Figures 3D, E**). Coincidently, the downregulation of Pax5 induced by LPS/COS treatment was inhibited (**Figure 4A**). Subsequently, we examined the influence of DNA methylation on B cell activation and found that the upregulation of Bcl6, Blimp1, and sIgM was compromised following AZA treatment, as revealed by qPCR analysis (**Figures 4B–D**). Furthermore, the enhancement of both antibody secreting ability and phagocytic ability of IgM^+^ B cells induced by LPS/COS treatment were compromised after AZA treatment (**Figures 4E–H**).

To further validate the influence of DNA methylation on Pax5 transcription, we performed *in vitro* methylation of pGL3-Pax5 plasmids using M. SssI methylase. The efficiency of DNA methylation was confirmed by digestion with the DNA methylation-sensitive enzyme *Hpa*II, which specifically digests unmethylated DNA (**Figure 4I**). Subsequently, the methylated or unmethylated plasmids were transfected into HEK293T cells and compared to a negative control (pGL3-Basic plasmid) to assess the promoter activity. The results indicated that methylation of the pGL3-Pax5 plasmids significantly repressed promoter activity of Pax5 (**Figure 4J**). Overall, our findings revealed a role of DNA methylation in B cell activation through inhibition of Pax5 expression.

## Discussion

In mammals, the development and maturation of B cells are precisely regulated by a cascade of orchestrated transcription factors. Among them, Pax5 serves as the key regulator of B cell development and maturation but is repressed during B cell activation. Although studies in mammals have shown that the plasma cell specific transcription factor Blimp1 is involved in the repression of Pax5, it remains unclear whether there are alternative mechanisms underlying its repression (11, 12).

As lower vertebrates, teleost also possess a relatively complete immune system. IgM^+^ B cells are a predominant subtype and play essential roles in systemic immune response. In this study, we established an efficient strategy for IgM^+^ B cell activation in large yellow croaker. COS, composed of deacetylated glucosamine and β- (1–4)-linked N-acetyl-D-glucosamine, exhibits beneficial effects in immunostimulatory, antibacterial and antioxidant activities and is widely used in drug delivery and tissue engineering (38). Previous studies have found that COS could effectively activate IgM^+^ B cells in grass carp (34). LPS, a component of the cell wall of Gram-negative bacteria, has been reported to be able to activate PKLs of large yellow croaker (19, 39). We found that the combined treatment with LPS and COS enhanced the phagocytic capacity, antibody secretion, and expression of activation-related genes of large yellow croaker IgM^+^ B cells. Interestingly, we found that the activation of B cells in large yellow croaker is accompanied with the suppression of Pax5 expression, suggesting that the repression of developmental related genes during B cell activation is conserved in teleost and mammals.

Previous studies have revealed that DNA methylation is involved in B cell development and activation. B cells at different developmental stages exhibit distinct DNA methylation levels (40). Conditional knockout of DNA demethylases Tet2/Tet3 at the pro-B cells stage impede its further differentiation into pre-B cells (41), while deletion of Tet2/Tet3 in mature B cells disrupts homeostasis and leads to tumorigenesis (42). We used a dot blot assay to examine the global DNA methylation level, and found that the B cell activation induced by LPS/COS is accompanied with a reduction in global DNA methylation level. Additionally, AZA treatment resulted in decreased global DNA methylation level both before and after B cell activation (**Supplementary Figure 1**). These results are consistent with the previous reports in mammals, where the majority of the differentially methylated genes during B cell activation were hypomethylated, while a small proportion of genes were hypermethylated (43). Interestingly, the deficiency of either DNA methyltransferase DNMT1 or DNA demethylase Tet2 leads to impaired B cell activation (43, 44), suggesting an essential and intricate role of DNA methylation during this process. In this study, we found that hypermethylation of CpG island1 in the promoter region of Pax5 is associated with its downregulation during B cell activation. Our results highlight the importance of examining downstream genes when exploring the role of DNA methylation. Future studies are required to elucidate whether DNA methylation collaborates with other factors, such as Blimp1, to regulate Pax5 expression.

## Data availability statement

The original contributions presented in the study are included in the article/**Supplementary Material**. Further inquiries can be directed to the corresponding author.

## Ethics statement

The animal study was approved by Committee on the Ethics of Animal Experiments of the Fujian Agriculture and Forestry University. The study was conducted in accordance with the local legislation and institutional requirements.

## Author contributions

YS: Conceptualization, Data curation, Investigation, Methodology, Software, Writing – original draft, Writing – review & editing. ZZ: Data curation, Investigation, Methodology, Writing – original draft. QC: Investigation, Methodology, Writing – original draft. XC: Conceptualization, Funding acquisition, Project administration, Resources, Supervision, Writing – review & editing.
