# Supplementary material for: DNA methylation regulates B cell activation via repressing Pax5 expression in teleost
Source: Front Immunol. 2024 Feb 9;15:1363426. doi: 10.3389/fimmu.2024.1363426 (PMC10884147; doi:10.3389/fimmu.2024.1363426)
Supplement: Supplementary file 1 [file DataSheet_1.docx]

**Supplementary Figure 1
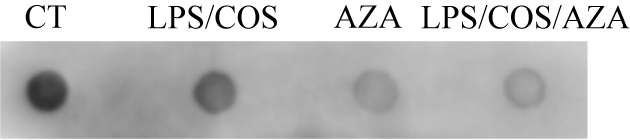
. Dot blot assay of global 5mC level during LPS/COS induced B cell activation and AZA treatment.** 500 ng of genomic DNA was used for dot blot.
